# RIFS: a randomly restarted incremental feature selection algorithm

**DOI:** 10.1038/s41598-017-13259-6

**Published:** 2017-10-12

**Authors:** Yuting Ye, Ruochi Zhang, Weiwei Zheng, Shuai Liu, Fengfeng Zhou

**Affiliations:** College of Computer Science and Technology, and Key Laboratory of Symbolic Computation and Knowledge Engineering of Ministry of Education, Jilin University, Changchun, Jilin, 130012 China

## Abstract

The advent of big data era has imposed both running time and learning efficiency challenges for the machine learning researchers. Biomedical OMIC research is one of these big data areas and has changed the biomedical research drastically. But the high cost of data production and difficulty in participant recruitment introduce the paradigm of “large p small n” into the biomedical research. Feature selection is usually employed to reduce the high number of biomedical features, so that a stable data-independent classification or regression model may be achieved. This study randomly changes the first element of the widely-used incremental feature selection (IFS) strategy and selects the best feature subset that may be ranked low by the statistical association evaluation algorithms, *e.g*. t-test. The hypothesis is that two low-ranked features may be orchestrated to achieve a good classification performance. The proposed Randomly re-started Incremental Feature Selection (RIFS) algorithm demonstrates both higher classification accuracy and smaller feature number than the existing algorithms. RIFS also outperforms the existing methylomic diagnosis model for the prostate malignancy with a larger accuracy and a lower number of transcriptomic features.

## Introduction

Modern biological technologies are rapidly revolutionized and improved in the recent years, and the biological OMIC data has been accumulated at an accelerated speed^1^. Human complex disease like cancers and cardiovascular diseases are known to be associated with more than one genetic factor^2,3^ and the classic single-factor correlation analysis tends to detect statistically significant factors^4^. So the existing complex disease diagnosis panels usually use the genetic information of multiple genes^5,6^.

The development of a disease diagnosis panel relies on the efficiency of the feature selection technologies^7^. A biological OMIC technology generates thousands or even millions of data entries for a single sample, but a biomedical project seldom recruited more than 1,000 samples due to various limitations, *e.g*. cost and patient availability^8^. The biomarker screening procedure may generate the overfit models due to the paradigm of “large *p*, small *n*”, where *p* and *n* are the numbers of features and samples, respectively^9,10^. Besides the aforementioned statistical reason, biomedical research results also show that not all the genes are biologically involved in a given disease onset and development processes^11^.

It is a computationally infeasible task to find a global optimal feature subset within a reasonable period^12^, and the existing feature selection algorithms may be roughly grouped as filter and wrapper approximate algorithms^13,14^. A filter algorithm evaluates each feature’s association with the class label using a statistical significance measurement^15^. Many biomedical biomarker were screened by the filter algorithms due to their linear time requirement, and sometimes is the only choice for large datasets like SNP and methylation polymorphisms^16,17^. But a filter algorithm only ranks the features by single-feature associations with the class labels, and the user is responsible for choosing the number of top-ranked features^18^. A wrapper algorithm evaluates each heuristically selected feature subset using a classification algorithm and tends to achieve better classification performance than the filters since a wrapper algorithm directly optimizes the target classification algorithm. A wrapper usually runs much slower than a filter algorithm, due to its consideration of inter-feature relationships^19^.

This study proposed a modified incremental feature selection strategy for the filter algorithms. An Incremental Feature Selection (IFS) algorithm evaluates the classification performance of the top-*k*-ranked features iteratively for *k* ∈ (1, 2, …, *n*), where *n* is the total number of features. IFS usually stops at the first observation of performance decrease^13,20^. This study proposes an IFS strategy by selecting features incrementally from a randomly-chosen starting feature and output the best solution from multiple Randomly re-started IFS (RIFS) procedures. The comparison with the existing filters and wrappers demonstrates that RIFS outperforms them by both higher classification accuracies and smaller feature numbers.

## Material and Methods

### Binary classification problem

This study evaluates a feature subset using the binary classification performance. A binary classification problem has two groups of samples, *i.e*. the Positive (*P*) and Negative (*N*) samples^13,21^. *P* and *N* are also used to denote the numbers of positive and negative samples. The binary classification problem is the simplest classification model, and usually a heuristic rule was employed to find the solution. And this is also the most widely adopted problem setting for biomedical researchers, *e.g*. disease versus control samples in the Genome-Wide Association Study (GWAS)^22^, and the samples of two phenotypes in the clinical survival analysis^23^, *etc*.

### Two groups of feature selection algorithms

This study compares the proposed algorithm with two major groups of feature selection algorithms, *i.e*. filters and wrappers^24–26^. Three filters, *i.e*. T-test based ranking (Trank)^27^, false positive classification rate (FPR)^28^, and Wilcoxon-test based ranking (Wrank)^29^, are evaluated when they select the same numbers of features as to the proposed algorithm in this study. Wrappers can directly recommend a list of features without the user-determined number of features^30^. Three wrappers, *i.e*. Lasso, Random Forest (RF) and Ridge Regression (Ridge), were compared with RIFS in this study. So this study investigated both the classification performances and the numbers of features for these feature selection algorithms. Two of the algorithms, Trank and Wrank, are from the Python scipy package, and all the other algorithms are from the Python scikit-learn package. Wrapper algorithms may achieve differently using different parameters. We assume that the default parameters of a wrapper algorithm should work well in most cases. To carry out a fair comparison, the RIFS’s parameters were optimized over four datasets ALL1/ALL2/ALL3/ALL4, and this study conducted the comprehensive comparison between RIFS and the existing algorithms on all the 17 transcriptome datasets. The Lasso parameter Alpha was set to 0.1. All the other parameters of feature selection algorithms utilized the default values.

### Performance measurements

A binary classification algorithm optimizes the parameters of a model and predicts that a new sample belongs to the positive (*P*) or negative (*N*) group. The sizes of the positive and negative groups are denoted as *P* and *N*, respectively. A positive sample is defined as a true positive or false negative one if it is predicted as positive or negative. And a negative sample is defined as a false positive or a true negative if its prediction is positive or negative. The numbers of true positives, false negatives, false positives and true negatives are denoted as *TP*, *FN*, *FP* and *TN*, respectively. The binary classification performance is evaluated by the following measurements, as similar in^13^. This study defines sensitivity (*Sn*) and specificity (*Sp*) as the percentages of correctly predicted positive and negative samples, *i.e. Sn* = *TP*/(*TP* + *FN*) and *Sp* = *TN*/(*TN* + *FP*). The overall accuracy is defined as *Acc* = (*TP* + *TN*)/(*TP* + *FN* + *TN* + *FP*). *F-score* is also known as *F*-*measure* or *F*_1_-*score* and has been widely used to evaluate the performance of a binary classification model^31^. *F-score* is defined as 2 × (*Precision* × *Sn*)/(*Precision* + *Sn*), and *Precision* = *TP/(TP* + *FP)*.

Five representative classification algorithms were evaluated on the datasets, and the maximal accuracy achieved by these five algorithms on a given feature subset of a dataset was defined as the maximal accuracy *mAcc*. Support Vector Machine (SVM) is a popular binary classification algorithm. K Nearest Neighbors (KNN) algorithm is an intuitive distance-based classification algorithm. Decision Tree (DTree) will generate an easy-to-interpret classifier. And Naïve Bayesian classifier (NBayes) assumes that all the features are independent to each other. Logistic Regression (LR) trains a linear classification function, which may suggest the weights of the chosen features.

All the algorithms were tested under two major Python releases, *i.e*. 2.7.13 and 3.6.0.

### Biomedical datasets

Data pre-processing is one of the most important steps for a data modeling problem. This study focused on the feature selection problem, and only checked the datasets for the issue of missing data. A feature was excluded from further analysis if it has missing data for some samples.

The proposed algorithm in this study was compared with the existing algorithms using 17 datasets, as similar in^14^. Each of the 17 datasets has two class labels, *i.e*. a binary classification problem. Six transcriptome datasets, *i.e*. DLBCL^32^, Pros^33^, ALL^34^, CNS^35^, Lym^36^ and Adeno^37^, were publicly available at the Broad Institute Genome Data Analysis Center. The two datasets *Colon* ^38^ and *Leuk* ^39^ were provided in the R/Bioconductor packages *colonCA* and *golubEsets*, respectively. The dataset ALL modeled as four binary classification datasets, *i.e*. ALL1/ALL2/ALL3/ALL4, based on different phenotype annotations, as described in Table 1. Five more recent datasets, *i.e*. Mye (accession: GDS531)^40^, Gas (accession: GSE37023)^41^, Gas1/Gas2 (accession: GSE29272)^42^, T1D (accession: GSE35725)^43^ and Stroke (accession: GSE22255)^44^, were publicly available at the NCBI Gene Expression Omnibus (GEO) database. The raw data from the NCBI GEO database was normalized into the gene expression matrix with the default parameters of the RMA algorithm^45^, and all the other datasets were downloaded as the normalized data matrix.

**Table 1:** Summary information of the 17 binary classification datasets.

ID	Dataset	Samples	Features	Summary
1	DLBCL	77	7,129	DLBCL (58) vs follicular lymphoma (19)
2	Pros	102	12,625	prostate cancer (52) vs control (50)
3	Colon	62	2,000	colon cancer (40) vs normal (22)
4	Leuk	72	7,129	ALL (47) vs AML (25)
5	Mye	173	12,625	presence (137) vs absence (36) of focallesions of bone
6	ALL1	128	12,625	B-cell (95) vs T-cell (33) ALL
7	ALL2	100	12,625	ALL with (65) vs without (35) relapse
8	ALL3	125	12,625	ALL with (24) vs without (101) multidrug resistance
9	ALL4	93	12,625	ALL with (26) and without (67) the t(9;22) chromosome translocation
10	CNS	60	7,129	medulloblastoma survivors (39) vs treatment failures (21)
11	Lym	45	4,026	germinalcentre (22) vs activated B-like DLBCL (23)
12	Adeno	36	7,457	colon adenocarcinoma (18) vs normal (18)
13	Gas	65	22,645	gastric cancer (29) vs non-malignants (36)
14	Gas1	144	22,283	non-cardia gastric cancer (72) vs normal (72)
15	Gas2	124	22,283	cardia gastric cancer (62) vs normal (62)
16	T1D	101	54,675	T1D (57) vs control (44)
17	Stroke	40	54,675	ischemic stroke (20) vs control (20)

Datasets 1–15 are the cancer transcriptomes, while the last two are transcriptome datasets of type I diabetes and stroke, respectively.

A recent study proposed that methylomes outperformed transcriptomic profiles in separating prostate cancers from the control samples^46^. We demonstrated that a good choice of transcriptomic features might also achieve a similarly good classification model compared with methylomes. The dataset GSE55599 was downloaded from the Gene Expression Omnibus (GEO) database^47^. The binary classification problem worked on the 32 prostate carcinoma samples and 10 benign prostatic hyperplasia samples. Each sample has 47,231 probesets, *i.e*. features.

### RIFS, a randomly re-started incremental feature selection algorithm

The incremental feature selection algorithm was modified to have a start position *k* and the consecutive performance decreasing cutoff *D*, which was denoted as the algorithm *sIFS*(*k*, *D*). For a binary classification problem with *n* features and *m* samples, the features are ranked based on their association significance with the binary class labels. A feature’s association significance with the class label is calculated by the statistical significance *P value* of the t-test^27^. Features are denoted as *f*_*i*_, *i* ∈ {1, 2, …, *n*}, based on their ranks. The algorithm will consecutively add the next element to the feature subset until the binary classification accuracy decreases consecutively *D* times. The pseudo-code of the algorithm *sIFS*(*k*, *D*) is shown as follows.
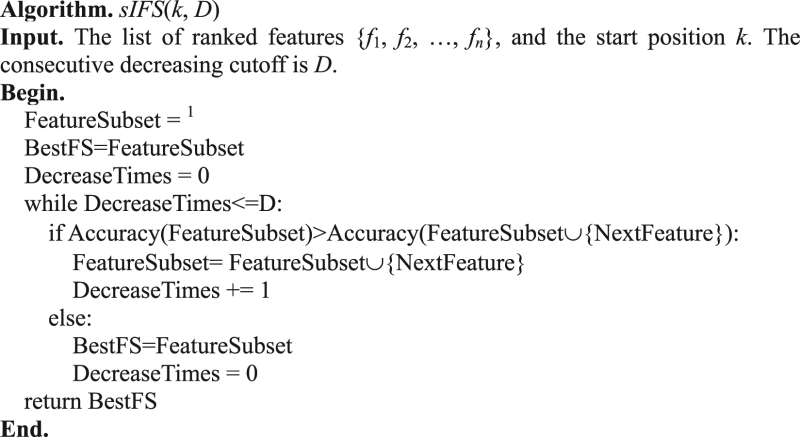


The Randomly re-started Incremental Feature Selection (RIFS) algorithm is proposed based on the unit algorithm *sIFS*(*k*, *D*). Our hypothesis is that a summarization of multiple *sIFS*(*k*, *D*) algorithms may generate a feature subset with better classification accuracy than the classical algorithms sIFS(1, 1). The pseudo-code of RIFS was shown as follows.
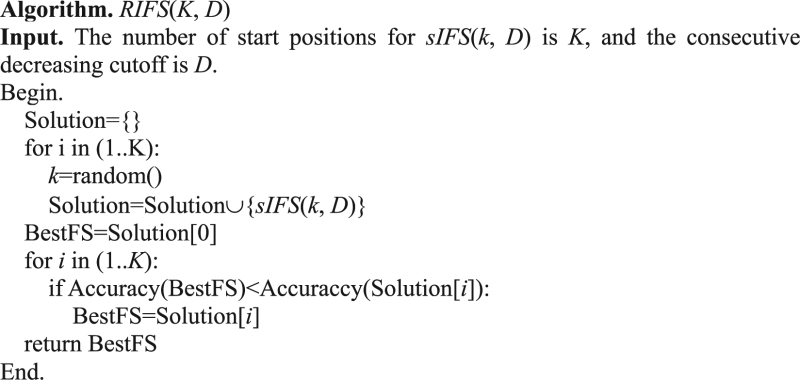


### Randomly seeded k-fold cross validation

A *k*-fold cross validation (KFCV) strategy is utilized to calculate the overall classification performance. The dataset was randomly split into *k* folds and used each fold to validate the model trained from the rest of *k*−1 folds. Given a binary classification dataset with positive and negative samples in the subsets *P* and *N*, respectively, KFCV randomly splits *P* and *N* into *k* equally-sized subsets, respectively. *P* = {*P*_1_, *P*_2_, …, *P*_*k*_} and *N* = {*N*_1_, *N*_2_, …, *N*_*k*_}. Iteratively, *P*_*i*_ ∪ *N*_*i*_ is selected as the testing dataset, and the other samples are used as the training data for a given classification algorithm. The classification performance measurements *Sn*, *Sp* and *Acc* are calculated based on this round of iteration.

RIFS selects features using the incremental rule from the given starting feature, and only the feature subset with the best classification performance will be kept for further analysis. Due to that different data splitting will generate different classification performances, multiple random seeds will be employed to produce KFCV calculations. The classification performance measurements are averaged over all the rounds of KFCV experiments. To eliminate the effects of over-fitting and random splitting, this study carried out 20 random runs of 10-fold cross-validation and chose the classification accuracy maximized over five classifiers, *i.e*. SVM, KNN, DTree, NBayes, and LR.

### Experimental procedure

**Figure 1:**
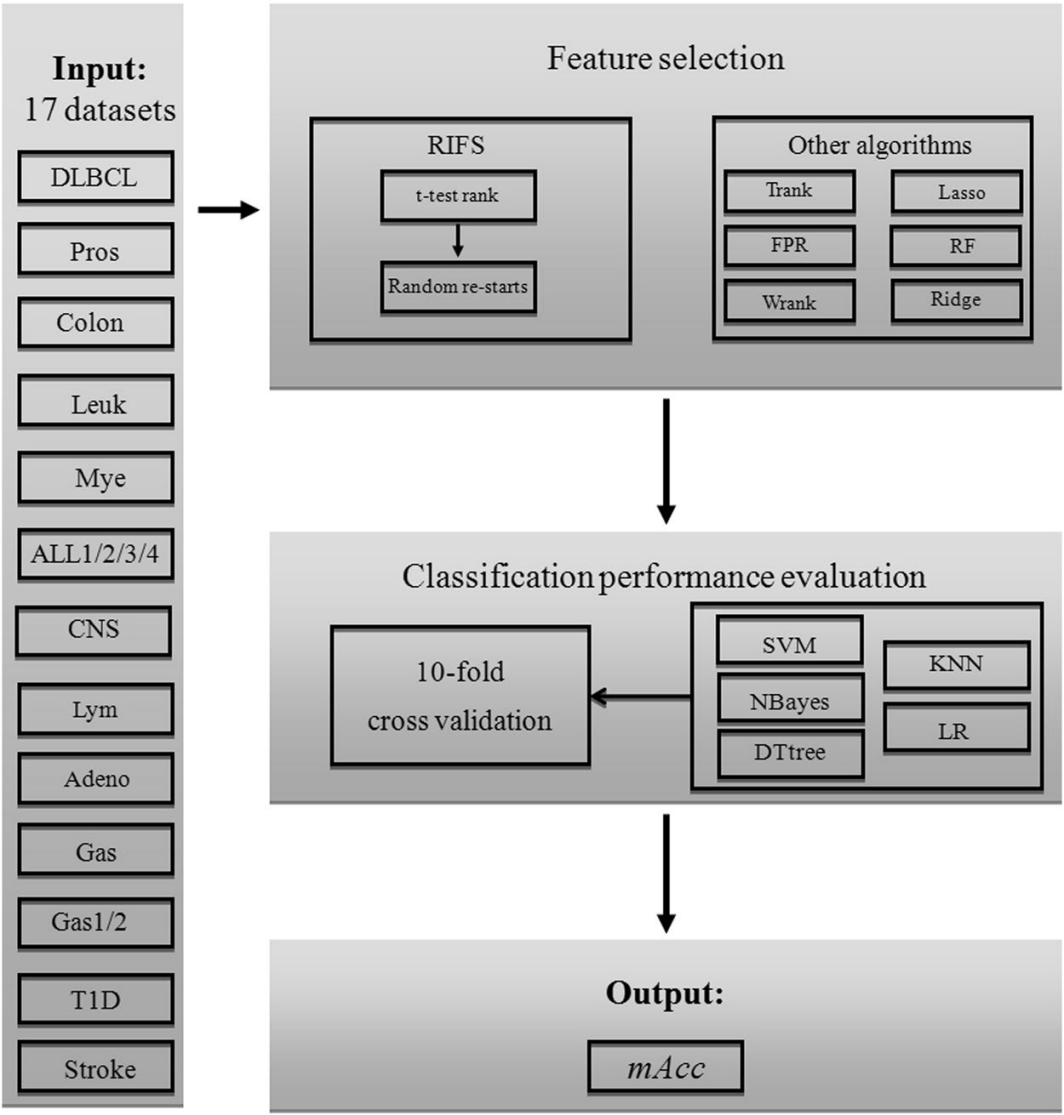
Experimental setting of this work. 17 datasets were chosen to compare RIFS with three filters and three wrappers, and the classification performances were calculated using 10-fold cross-validations.

RIFS was compared with two major classes of feature selection algorithms, *i.e*. three filters and three wrappers. The performance measurement was calculated using 10-fold cross-validation, and the classification accuracy was the maximum value *mAcc* of the five classification algorithms, *i.e*. SVM, KNN, DTree, NBayes and LR. The detailed procedure was illustrated in Fig. 1.

All the experiments were carried out in an Inspur Gene Server G100, with 256GB memory, 28 Intel Xeon® CPU cores (2.4 GHz), and 30TB RISC1 disk space.

## Results and Discussion

### Two optimization rules for the IFS strategy

**Figure 2:**
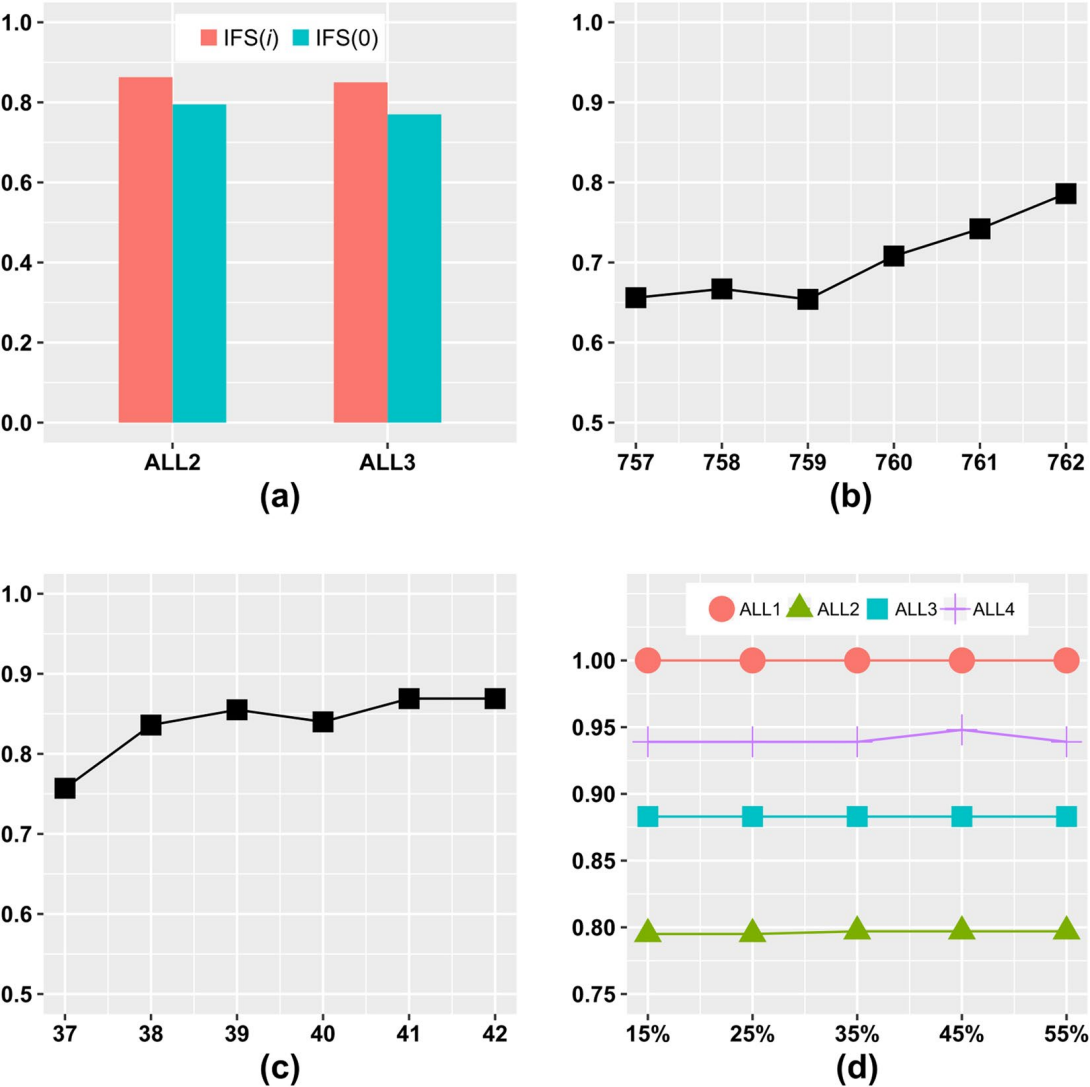
Demonstrative examples of RIFS rules and evaluation of the best starting percentage. (**a**) Two features starting from the rank *i* = 31 for the dataset ALL2. 4 features starting from the rank *i* = 443 for the dataset ALL3. (**b**) Accuracy curve of IFS(757) for the dataset T1D. (**c**) Accuracy curve of IFS(37) for the dataset Colon. (**d**) The maximum accuracy is calculated for each of the four datasets, *i.e*. ALL1/ALL2/ALL3/ALL4, with different percentages of all the features as the starting points.

This study proposes two hypothetical modifications of the Incremental Feature Selection (IFS) strategy^13,20,48^ based on the experiment data, as shown in Fig. 2. The optimization goal is to maximize the binary classification accuracy using the selected feature subset. The classification performance in this demonstration step was calculated by one round of 10-fold cross validation.

#### Randomly re-start the IFS strategy

We generalize the classical version of IFS as the IFS(*i*), which chooses a subset of consecutively ranked features starting from the rank *i*. Our hypothesis is that there may exist a feature subset IFS(*i*) with a classification accuracy better than IFS(0). This hypothesis is supported by the two examples in Fig. 2(a). The two features with the ranks 31 and 32 achieved 79.5% in the overall accuracy for the dataset ALL2, better than 77.0% in accuracy for the top two ranked features by IFS(0), as shown in Fig. 2. The four features with the ranks 443, 444, 445 and 446 outperformed the top four ranked features by 1.3% in accuracy for the dataset ALL3. Their statistical significances measured in *P-values* are 0.036990, 0.036994, 0.037100 and 0.037105 for these four low-ranked features, respectively. So the IFS strategy may be improved by the randomly re-starting rule.

#### Stop at one decrease does not work well

We also observe that a big increase in the classification performance may be achieved by adding two consecutively ranked features, even when there is a decrease by adding the first one. For example, the feature screening process IFS(37) got the first accuracy decrease (1.5%) for the dataset Colon when adding the four^th^ feature (ranked 40). But IFS(37) achieved an increase (1.4%) in accuracy even compared with the situation before adding the four^th^ feature, as shown in Fig. 2(b). Another case was the feature screening process IFS(757) for the dataset T1D, as in Fig. 2(c). The integration of the third feature decreased the accuracy by 1.3%, but the next feature (ranked 760) increased the accuracy by 5.4%, which was also higher than the two consecutively ranked features 757 and 758 by 4.1% in accuracy. So the stop strategy of IFS(*i*) needs to tolerate at least one accuracy decrease.

### How many starting points are enough for most datasets?

We investigated the best number of starting points for RIFS using the four datasets, ALL1/ALL2/ALL3/ALL4, as shown in Fig. 2(d). RIFS was set to stop if three consecutive tries do not increase the classification performances. Five choices of the numbers of starting points were evaluated, *i.e. pStartingPercentage* = 15%, 25%, 35%, 45%, 55% of the total feature number, respectively. The classification performance in this parameter optimization step was calculated by one round of 10-fold cross validation. The classification algorithms achieved 100% in *mAcc* for all the six values of the parameter *pStartingPercentage* on the dataset ALL1. It seems that the dataset ALL1 is easy to separate, and some other algorithms also achieved 100% in *mAcc*, as demonstrated in^14^. The measurement *mAcc* for the dataset ALL2 was improved from 79.5–80.6% when the parameter *pStartingPercentage* increased from 15–55%, and the maximum value 80.6% was achieved after *pStartingPercentage* = 45%. The measurement *mAcc* remained 88.3% for all different values of *pStartingPercentage* for the dataset ALL3. And the best *mAcc* = 94.9% was achieved after *pStartingPercentage* = 45%. So the default value 45% was set for the parameter *pStartingPercentage*.

### How much tolerance for consecutive performance decreases is enough?

A greedy feature selection algorithm tends to stop when the optimization goal decreases during the feature screening process, *e.g*. the classical IFS strategy. Our hypothesis is that after a minor decrease in the classification performance, adding the next feature may achieve a much better overall performance improvement.

**Figure 3:**
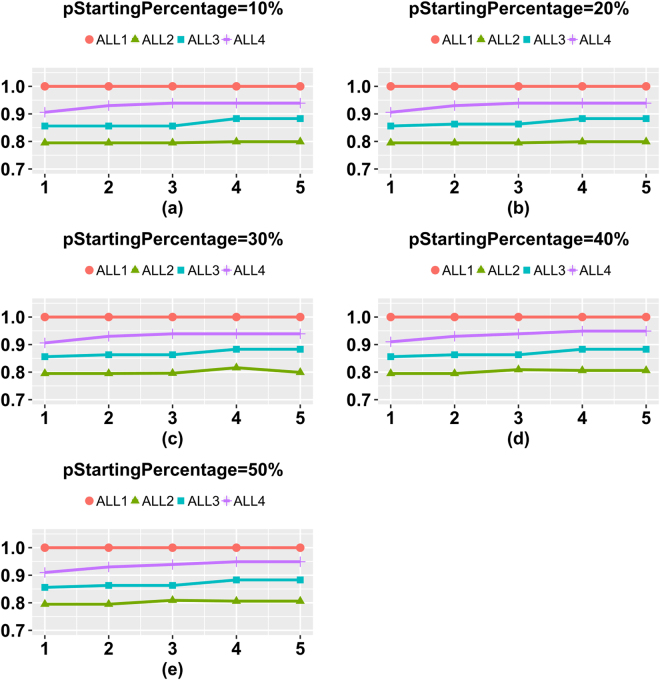
How many steps are tolerated without performance improvements. The classification performance is measured in *mAcc*.

We evaluated the RIFS stopping criteria *pStoppingDepth* = 1, 2, 3, 4 and 5, *i.e*. RIFS stops when *pStoppingDepth* consecutive performance decreases are detected. The four datasets ALL1/ALL2/ALL3/ALL4 were chosen for the evaluation. The parameter *pStartingPercentage* was set to 10%, 20%, 30%, 40% and 50%, and the performance measurement is *mAcc* by the 10-fold cross validation strategy. The classification performance in this parameter optimization step was calculated by one round of 10-fold cross validation. Figure 3 demonstrated that RIFS achieved the best *mAcc* when *pStoppingDepth* reached 4 for all the four datasets using the 5 values of *pStartingPercentage*. So the experimental data supported our hypothesis that it may not be the best choice to stop immediately after one performance decrease was detected. And the default value of *pStoppingDepth* was chosen as 4.

**Figure 4:**
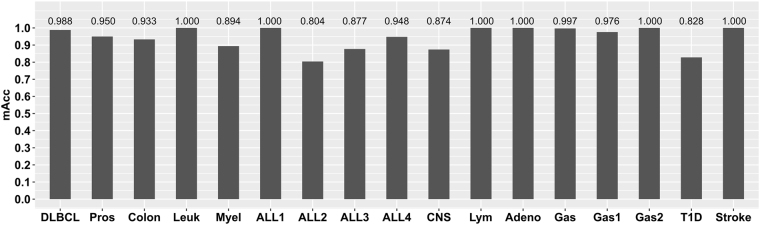
The classification performances of RIFS on the 17 transcriptome datasets. The measurement *mAcc* is used as the vertical axis, and the horizontal axis lists the 17 datasets. The detailed mAcc values are also given on the top of each column.

A comprehensive evaluation of RIFS with the default parameter values *pStartingPercentage* = 45% and *pStoppingDepth* = 4 was carried out for all the 17 transcriptome datasets, as shown in Fig. 4. The classification performance in the following comparative analysis steps was calculated by 20 random rounds of 10-fold cross validation. RIFS achieved at least 0.804 in *mAcc* for these datasets, and even achieved 1.000 in *mAcc* for 6 out of the 17 datasets. The following sections will compare RIFS with the existing feature selection algorithms by the performance measurement *mAcc*.

### Comparison with filters on the 17 transcriptomes

RIFS with the default parameter values *pStartingPercentage* = 45% and *pStoppingDepth* = 4 was compared with the filter algorithms. A filter algorithm assumes that features are independent to each other, and evaluates the association of a feature with the class label independently. So users need to determine how many features will be chosen, after all the features are evaluated and ranked by the filter algorithm. In order to conduct a fair comparison, if RIFS selects *k* features, this study selects the same number *k* of top-ranked features evaluated by a filter. 10-fold cross-validation strategy was employed to calculate the binary classification performances of RIFS and the three filter algorithms, *i.e*. Trank, FPR, and Wrank. RIFS improves the feature selection procedure based on a filter algorithm, so it is anticipatable that RIFS outperforms the filter algorithms.

**Figure 5:**
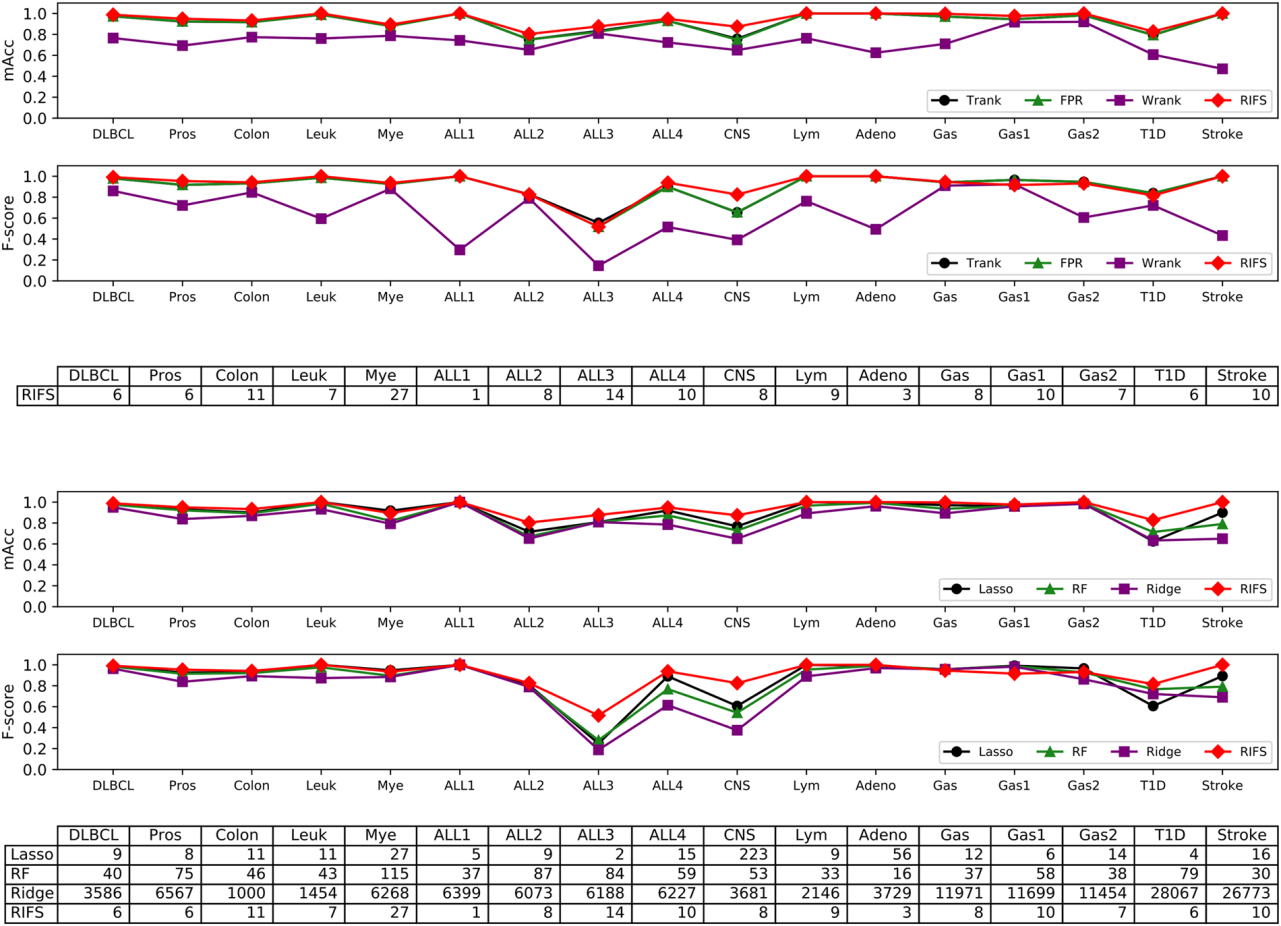
Performance comparison of RIFS with 3 filters and 3 wrappers. The vertical axis is the performance measurement *mAcc* and F-score, and the horizontal axis gives the dataset names. Since all the filter algorithms select the same number of features as RIFS, only the numbers of features by RIFS are shown. The last table gives the numbers of features selected by the wrappers algorithms and RIFS. (**a**) Comparison with 3 filters. (**b**) Comparison with 3 wrappers.

RIFS performed the best compared with the three filter algorithms on all the 17 datasets, as shown in Fig. 5(a). The four datasets ALL1, Lym, Adeno and Stroke, seem to be easy to be separated, since three feature selection algorithms including RIFS achieved 100% in *mAcc*. RIFS outperformed the three filter algorithms on all the other 13 datasets. And CNS seems to be a difficult binary classification dataset. RIFS achieved 87.4% in *mAcc*, and improved the other filter algorithms by at least 11.6% in *mAcc*. RIFS usually selects no more than 10 features, and the maximal number of features selected by RIFS was 27 for the dataset Mye.

The experimental data suggests that an orchestration of low-ranked features may achieve very good classification performances even for the difficult datasets like CNS and ALL2. No filter algorithms achieved *mAcc* better than 76.0%, and RIFS only achieved 80.4% and 87.4% in *mAcc* for the datasets ALL2 and CNS, respectively.. This provides an additional piece of evidence for the rule “Randomly re-start the IFS strategy” of RIFS.

RIFS achieved F-score >  = 0.900 on 13 out of the 17 transcriptome datasets, and the maximal F-scores on 12 datasets compared with the 3 filters, as shown in Fig. 5(a). The maximal F-score improvement 0.049 compared with RIFS’s F-score = 0.916 was achieved by Trank and FPR on the dataset Gas1. The next biggest F-score improvement 0.038 compared with RIFS was achieved by Trank on the dataset ALL3. The data suggested that ALL3 was a difficult dataset for the filter algorithms.

### Comparison with wrappers on the 17 transcriptomes

RIFS with the default parameters *pStartingPercentage* = 45% and *pStoppingDepth* = 4 performed the best compared with the three wrapper algorithms, *i.e*. Lasso, RF and Ridge, on all the transcriptome datasets except for Myel, as also shown in Fig. 5(b). RIFS achieved 100% in *mAcc* for 6 out of the 17 datasets, and its average *mAcc* is 94.5%. The next best algorithm based on the average *mAcc* is Lasso. Lasso achieved the average *mAcc* 90.4% and *mAcc* = 100% for the three datasets Leuk, ALL1 and Lym. Lasso was also the only wrapper algorithm outperforming RIFS with 2.4% in *mAcc* on the dataset Mye. Except for this case, RIFS performed better than all the wrapper algorithms on all the datasets, and achieved an average improvement 3.5% in *mAcc* for the best of the three wrapper algorithms on the 17 transcriptomic datasets.

Another performance measurement for a feature selection algorithm is the number of features selected by the algorithm. Besides the excess consumption of computational power in training and predicting by a classification model with a large number of features, the overfitting problem is also inevitable to be fixed^49^. Due to the high data production cost in the biomedical area, the number of samples is usually much smaller than that of features in a biomedical dataset^50^. And the final clinical deployment of a classification model has a cost positively correlated with the number of features in the model. So a biomedical classification model with a higher accuracy and a smaller number of features is preferred in the clinical settings.

RIFS recommended a smaller list of features for training classification models with higher accuracy except for six cases, as shown in the table under the line plot in Fig. 5. Both RIFS and Lasso recommended 11 features for the dataset Colon, but RIFS outperformed Lasso with an improvement 3.3% in *mAcc*. Lasso selected the same number of features as RIFS for the dataset Lym, and both achieved 100% in *mAcc*. And Lasso outperformed RIFS with an improvement 2.4% in *mAcc* on the dataset Mye. For the three datasets ALL3, Gas1 and T1D, Lasso recommended fewer features than RIFS, but RIFS achieved improvements in *mAcc* 6.7%, 1.5% and 11.4%, respectively. Overall, RIFS suggested an average number of features 8.882, while the three wrapper algorithms chose 25.706, 54.706, and 8428.353 features, respectively.

RIFS achieved the maximal F-score on 13 out of the 17 transcriptome datasets compared with the 3 wrapper algorithms. Except for the dataset ALL3, RIFS achieved at least 0.800 in F-score on all the other 16 datasets. The maximal F-score improvement 0.076 compared with RIFS was achieved by the algorithm Lasso on the dataset Gas1. RIFS didn’t work well on the three gastric cancer datasets Gas/Gas1/Gas2 and the dataset Mye compared with the 3 wrappers. RIFS achieved the biggest F-score improvement 0.237 on the dataset ALL3 compared with the three wrappers.

### Transcriptome performs similarly well with methylome

**Figure 6:**
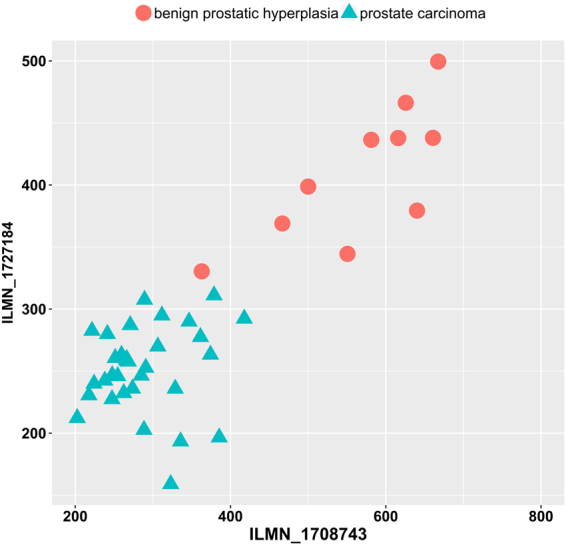
Dot plot of the two features detected by RIFS. There is only one benign prostatic hyperplasia sample which is very close to the prostate carcinoma ones.

RIFS was employed to screen the transcriptomes of prostate samples and detected two features with 100% discrimination accuracy of prostate cancers. A recent study suggested that top-ranked methylome features outperformed the top-ranked transcriptome features by at least 5.7% in the measurement Area Under the ROC Curve (AUC)^46^. A combination of three methylome features achieved 100% in both the overall accuracy and AUC, while three expression features only achieved 0.978 in AUC. RIFS detected two features ILMN_1708743 and ILMN_1727184 as the biomarkers to discriminate the samples of prostate carcinoma and benign prostatic hyperplasia, as illustrated in Fig. 6. We may see that these two biomarkers can easily separate the 32 prostate carcinoma and ten benign prostatic hyperplasia samples. There is only one sample which is very close to the prostate carcinoma samples in the two-dimensional plane. And a non-linear-kernel SVM can achieve 100% in *mAcc* for this binary classification problem.

### How random seeds impact the feature subsets?

Different random seeds generate different series of random numbers, so we evaluated how random seeds affect the performance of RIFS. To conduct a fair comparison, RIFS was run with the default random seed 0 in the above experiments. This section executed RIFS on the dataset Mye using integers from 0 to 20 as the random seeds. Default values were chosen for the two RIFS parameters *pStartingPercentage* = 45% and *pStoppingDepth* = 4. Table 2 shows that the random seeds 1/4/10/11/14/15/19 generated the same feature subset as the random seed 0, and the 27 features achieved the best mAcc = 89.8%. And the random seeds 3/13/16/18 even found a feature subset of 13 features and achieved 89.0% in mAcc. So if a user may prefer a smaller feature subset, multiple tries of different random seeds are recommended.

**Table 2:** Results of RIFS with different random seeds.

**Random seeds**	**Rank**	**NumF**	**mAcc**
1,2,4,10,11,14,15,19	379	27	89.8%
3,13,16,18	179	13	89.0%
5,6,9,12,20	66	17	89.2%
7	1	18	88.7%
8,17	63	21	89.1%

The first column gives the random seeds. Some random seeds generated the same feature subset, so they were summarized in the same row. The columns Rank, NumF and mAcc give the rank of the first feature, the feature number and the classification measurement mAcc of the best feature subset.

## Conclusions

RIFS demonstrated a new perspective of feature selection that two individually low-ranked features might work together to make a highly accurate classification model. RIFS can detect features with accurate classification performances, by significantly expanding the searching space. RIFS also tries to avoid the local optimal solutions by tolerating more than one classification performance decreases. There is a balance between the running time and the classification performance, but the user has the flexibility of choosing better classification accuracy for a long running time or an acceptable accuracy within a short period.

## References

[1] Stephens ZD (2015). Big Data: Astronomical or Genomical?. PLoS biology.

[2] Dai X, Xiang L, Li T, Bai Z (2016). Cancer Hallmarks, Biomarkers and Breast Cancer Molecular Subtypes. Journal of Cancer.

[3] Selvaraju V (2012). Diabetes, oxidative stress, molecular mechanism, and cardiovascular disease–an overview. Toxicology mechanisms and methods.

[4] Atanasovska B, Kumar V, Fu J, Wijmenga C, Hofker MH (2015). GWAS as a Driver of Gene Discovery in Cardiometabolic Diseases. Trends in endocrinology and metabolism: TEM.

[5] Figueroa JD (2014). Genome-wide interaction study of smoking and bladder cancer risk. Carcinogenesis.

[6] Cuperlovic-Culf M, Belacel N, Davey M, Ouellette RJ (2010). Multi-gene biomarker panel for reference free prostate cancer diagnosis: determination and independent validation. Biomarkers: biochemical indicators of exposure, response, and susceptibility to chemicals.

[7] Baek S, Tsai CA, Chen JJ (2009). Development of biomarker classifiers from high-dimensional data. Briefings in bioinformatics.

[8] Tomczak K, Czerwinska P, Wiznerowicz M (2015). The Cancer Genome Atlas (TCGA): an immeasurable source of knowledge. Contemporary oncology.

[9] Sanchez BN, Wu M, Song PX, Wang W (2016). Study design in high-dimensional classification analysis. Biostatistics.

[10] Shujie MA, Carroll RJ, Liang H, Xu S (2015). Estimation and Inference in Generalized Additive Coefficient Models for Nonlinear Interactions with High-Dimensional Covariates. Annals of statistics.

[11] Li Y, Patra JC (2010). Genome-wide inferring gene-phenotype relationship by walking on the heterogeneous network. Bioinformatics.

[12] Yusta SC (2009). Different metaheuristic strategies to solve the feature selection problem. Pattern Recognition Letters.

[13] Guo P (2014). Gene expression profile based classification models of psoriasis. Genomics.

[14] Ge R (2016). McTwo: a two-step feature selection algorithm based on maximal information coefficient. BMC bioinformatics.

[15] Radovic M, Ghalwash M, Filipovic N, Obradovic Z (2017). Minimum redundancy maximum relevance feature selection approach for temporal gene expression data. BMC bioinformatics.

[16] Ciuculete DM (2017). A methylome-wide mQTL analysis reveals associations of methylation sites with GAD1 and HDAC3 SNPs and a general psychiatric risk score. Translational psychiatry.

[17] Lin H (2017). Methylome-wide Association Study of Atrial Fibrillation in Framingham Heart Study. Scientific reports.

[18] Gardeux V (2015). Computing molecular signatures as optima of a bi-objective function: method and application to prediction in oncogenomics. Cancer informatics.

[19] Yu L, Liu H (2004). Efficient feature selection via analysis of relevance and redundancy. Journal of machine learning research.

[20] Chen W, Ding H, Feng P, Lin H, Chou KC (2016). iACP: a sequence-based tool for identifying anticancer peptides. Oncotarget.

[21] Zhou F, Xu Y (2010). cBar: a computer program to distinguish plasmid-derived from chromosome-derived sequence fragments in metagenomics data. Bioinformatics.

[22] Chapuis, J. *et al*. Genome-wide, high-content siRNA screening identifies the Alzheimer’s genetic risk factor FERMT2 as a major modulator of APP metabolism. *Acta neuropathologica*, doi:10.1007/s00401-016-1652-z (2016). 10.1007/s00401-016-1652-z PMC5427165 27933404

[23] Shirahata M (2007). Gene expression-based molecular diagnostic system for malignant gliomas is superior to histological diagnosis. Clinical cancer research: an official journal of the American Association for Cancer Research.

[24] Dash M, Liu H (1997). Feature selection for classification. Intelligent data analysis.

[25] Guyon I, Elisseeff A (2003). An introduction to variable and feature selection. The Journal of Machine Learning Research.

[26] Liu H, Yu L (2005). Toward integrating feature selection algorithms for classification and clustering. Knowledge and Data Engineering, IEEE Transactions on.

[27] Baldi P, Long AD (2001). A Bayesian framework for the analysis of microarray expression data: regularized t -test and statistical inferences of gene changes. Bioinformatics.

[28] Guyon I, Elisseeff A (2003). An introduction to variable and feature selection. Journal of machine learning research.

[29] Liu WM (2002). Analysis of high density expression microarrays with signed-rank call algorithms. Bioinformatics.

[30] Kohavi R, John GH (1997). Wrappers for feature subset selection. Artificial intelligence.

[31] Lipton ZC, Elkan C, Naryanaswamy B (2014). Optimal Thresholding of Classifiers to Maximize F1 Measure. . Machine learning and knowledge discovery in databases: European Conference, ECML PKDD…: proceedings. ECML PKDD.

[32] Shipp MA (2002). Diffuse large B-cell lymphoma outcome prediction by gene-expression profiling and supervised machine learning. Nature medicine.

[33] Singh D (2002). Gene expression correlates of clinical prostate cancer behavior. Cancer cell.

[34] Chiaretti S (2004). Gene expression profile of adult T-cell acute lymphocytic leukemia identifies distinct subsets of patients with different response to therapy and survival. Blood.

[35] Pomeroy SL (2002). Prediction of central nervous system embryonal tumour outcome based on gene expression. Nature.

[36] Alizadeh AA (2000). Distinct types of diffuse large B-cell lymphoma identified by gene expression profiling. Nature.

[37] Notterman DA, Alon U, Sierk AJ, Levine AJ (2001). Transcriptional gene expression profiles of colorectal adenoma, adenocarcinoma, and normal tissue examined by oligonucleotide arrays. Cancer Res.

[38] Alon U (1999). Broad patterns of gene expression revealed by clustering analysis of tumor and normal colon tissues probed by oligonucleotide arrays. Proceedings of the National Academy of Sciences of the United States of America.

[39] Golub TR (1999). Molecular classification of cancer: class discovery and class prediction by gene expression monitoring. Science.

[40] Tian E (2003). The role of the Wnt-signaling antagonist DKK1 in the development of osteolytic lesions in multiple myeloma. The New England journal of medicine.

[41] Wu YH (2013). Comprehensive genomic meta-analysis identifies intra-tumoural stroma as a predictor of survival in patients with gastric cancer. Gut.

[42] Wang, G. S. *et al*. Comparison of Global Gene Expression of Gastric Cardia and Noncardia Cancers from a High-Risk Population in China. *Plos One* **8** (2013). 10.1371/journal.pone.0063826 PMC3661768 23717493

[43] Levy H (2012). Transcriptional signatures as a disease-specific and predictive inflammatory biomarker for type 1 diabetes. Genes Immun.

[44] Krug T (2012). TTC7B emerges as a novel risk factor for ischemic stroke through the convergence of several genome-wide approaches. J Cerebr Blood F Met.

[45] Irizarry RA (2003). Exploration, normalization, and summaries of high density oligonucleotide array probe level data. Biostatistics.

[46] Paziewska A (2014). DNA methylation status is more reliable than gene expression at detecting cancer in prostate biopsy. British journal of cancer.

[47] Clough E, Barrett T (2016). The Gene Expression Omnibus Database. Methods in molecular biology.

[48] Chen L, Zhang YH, Huang T, Cai YD (2016). Gene expression profiling gut microbiota in different races of humans. Scientific reports.

[49] Lumbreras B (2009). Sources of error and its control in studies on the diagnostic accuracy of “‐omics” technologies. PROTEOMICS-Clinical Applications.

[50] Kosorok MR, Ma S (2007). Marginal asymptotics for the “large p, small n” paradigm: with applications to microarray data. The Annals of Statistics.

